# Hepatic Encephalopathy and Spontaneous Bacterial Peritonitis Improve Cirrhosis Outcome Prediction: A Modified Seven-Stage Model as a Clinical Alternative to MELD

**DOI:** 10.3390/jpm10040186

**Published:** 2020-10-22

**Authors:** Chien-Hao Huang, Hsiao-Jung Tseng, Piero Amodio, Yu-Ling Chen, Sheng-Fu Wang, Shang-Hung Chang, Sen-Yung Hsieh, Chun-Yen Lin

**Affiliations:** 1 Division of Hepatology, Department of Gastroenterology and Hepatology, Chang-Gung Memorial Hospital, Linkou Medical Center, Taoyuan City 33305, Taiwan; huangchianhou@gmail.com (C.-H.H.); Shanelily@msn.com (S.-F.W.); chunyenlin@gmail.com (C.-Y.L.); 2 College of Medicine, Chang-Gung University, Taoyuan City 33305, Taiwan; afen.chang@gmail.com; 3 Graduate Institute of Clinical Medical Sciences, College of Medicine, Chang-Gung University, Taoyuan City 33305, Taiwan; 4 Biostatistics Unit, Clinical Trial Center, Chang Gung Memorial Hospital, Linkou Medical Center, Taoyuan City 33305, Taiwan; allebjht@gmail.com; 5 Department of Medicine, University of Padova, 35122 Padova, Italy; piero.amodio@unipd.it; 6 Center for Big Data Analytics and Statistics, Department of Medical Research and Development, Chang Gung Memorial Hospital, Linkou Medical Center, Taoyuan City 33305, Taiwan; chenyuling0722@gmail.com; 7 Department of Cardiology, Chang-Gung Memorial Hospital, Linkou Medical Center, Taoyuan City 33305, Taiwan

**Keywords:** clinical stage models, MELD, cirrhosis, overall mortality, hepatic encephalopathy, spontaneous bacterial peritonitis

## Abstract

Classification of cirrhosis based on clinical stages is rapid and based on five stages at present. Two other relevant events, hepatic encephalopathy (HE) and spontaneous bacterial peritonitis (SBP), can be considered in a clinical perspective but no study has implemented a seven-stage classification and confirmed its value before. In addition, long-term validation of the Model for End-Stage Liver Disease (MELD) in large cohorts of patients with cirrhosis and comparison with clinical findings are insufficient. Therefore, we performed a study to address these items. From the Chang-Gung Research Database (CGRD), 20,782 patients with cirrhosis were enrolled for an historical survival study. The MELD score, the five-stage clinical score (i.e., occurrence of esophageal varices (EV), EV bleeding, ascites, sepsis) and a novel seven-stage clinical score (i.e., occurrence of EV, EV bleeding, ascites, sepsis, HE, SBP) were compared with their Cox models by receiver operating characteristic (ROC) analysis. The addition of HE and SBP to the seven-stage model had a 5% better prediction result than the five-stage model did in the survival ROC analysis. The result showed that the seven clinical stages are associated with an increased risk for mortality. However, the predicted performances of the seven-stage model and MELD system are likely equivalent. In conclusion, the study (i) proved that clinical staging of cirrhosis based on seven items/stages had higher prognostic value than the five-stage model and (ii) confirmed the validity of the MELD criteria vs. clinical assessment.

## 1. Introduction

Cirrhosis is the leading cause of liver-related death worldwide [1,2]. It is also currently ranked the 11th global leading cause of death [3]. The natural progression of cirrhosis is characterized by an asymptomatic phase, termed “compensated cirrhosis”, followed by a rapid progressive phase termed “decompensated cirrhosis” [4]. Decompensated cirrhosis is marked by the development of complications including variceal bleeding, ascites, hepatic encephalopathy (HE), and spontaneous bacterial peritonitis (SBP) [5]. The survival time of patients with decompensated cirrhosis is significantly shorter than that of compensated patients, with median survival times of 2 years and 12 years, respectively [4]. Once decompensated cirrhosis develops, the definitive treatment for patients is liver transplantation [6]. Since healthcare resources may be limited, it is important to predict mortality from cirrhosis, especially when planning the optimal timing of liver transplantation and/or other interventions [7].

Many prognostic models have been proposed to predict mortality from cirrhosis, including the widely used Child–Pugh score [8], and a model for end-stage liver disease (MELD) [9]. The MELD has been adopted by the United Network for Organ Sharing (UNOS) since 2002 to better prioritize liver transplantation (LT) waiting lists in the United States and other countries worldwide [10]. It can accurately predict three-month mortality among patients with chronic liver disease on the waiting list (Area under Receiver Operating Characteristic (AUROC) 0.83) [11]. One study found that allocation of donor livers based on the MELD rather than clinical judgment would significantly reduce 20% waiting list mortality [12]. by 15%. The biggest advantage of the MELD is that it is based on multivariable analysis of objective tests for serum bilirubin, international normalized ratio (INR), and serum creatinine, thereby minimizing subjective judgment by a clinician [13].

However, some limits of the MELD cannot be neglected: (i) its ability in predicting mortality beyond three months is not well validated [14,15], (ii) some variables used for its computations can be altered by extra-hepatic factors such as age, body weight, treatment, sepsis, or hemolysis [16], (iii) the role of clinical manifestations of cirrhosis is not reflected by the MELD score; thus, patients with disabling HE and/or ascites are not considered for liver transplantation in an adequate manner, unless exceptions to the MELD are used for organ allocation [17].

An alternative to the MELD, a criterion much more related to the clinical development of cirrhosis, was proposed by D’Amico et al. [4], with stages defined by the presence or absence of complications [18] with a markedly different prognosis. A simple and widely used five-stage model based on the presence of esophageal varices (EVs), ascites, EV bleeding, or sepsis with an estimated one-year mortality rate for each step was later implemented by Arvaniti [19]. Of note, this model did not consider either SBP or HE in predicting one-year mortality. The rate of SBP was higher than that of bacteremia (66.2% vs. 49.4%) [19]. Additionally, overt HE was an index of poor prognosis, requiring a timely orthotopic liver transplantation [20].

Moreover, it is worth noting that (i) improved survival has been observed in patients with variceal hemorrhage in recent years [21], (ii) patients with ascites may not receive adequate priority in transplant lists [6], (iii) a clinical stage prognostic model was addressed in the Baveno consensus [18]. We proposed a novel seven-complication prediction model including SBP and overt HE to assess if it improves the five-complication prediction model, and to compare the effectiveness with the MELD, which is well applied in clinical practice.

## 2. Materials and Methods

### 2.1. Data Source and Patient Selection

Data were obtained from Chang-Gung Research Database (CGRD) upon IRB approval (201701810BC101, 201802088B0). The CGRD is based on the largest healthcare system in Taiwan, which comprises two medical centers, two regional hospitals, and three district hospitals located from the northeast to southern regions of Taiwan [22]. The CGRD not only includes outpatient, emergency, and inpatient claim records, but also contains laboratory, endoscopic, microbiological, and image reports and drugs. More detailed information about CGRD has been reported in a previous article [23]. Owing to the big database, statistical analysis may make the results more reliable.

**Figure 1:**
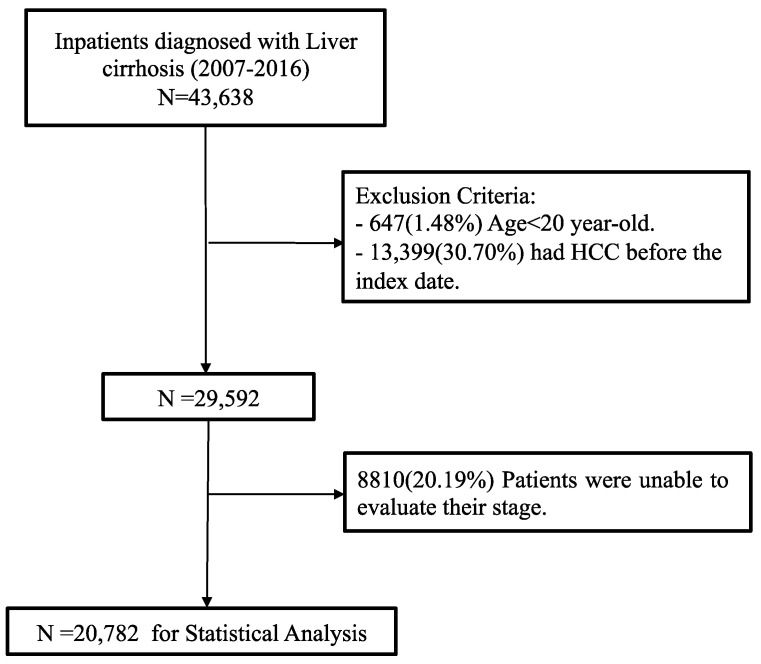
The flowchart for enrollment.

The data we analyzed were collected between 2007 and 2016. The inclusion criterion were patients diagnosed with definite liver cirrhosis (LC) confirmed by the International Classification of Diseases, Ninth Revision, Clinical Modification (ICD-9-CM) diagnosis code (571.2, 571.5, 571.6, 572.3) or 10th Revision (ICD-10) code: K70.3, K71.7, K74 (K740~K74.6), K76.6 and an abdominal echography report. Exclusion criteria included previous diagnosis of hepatocellular carcinoma before enrollment, age <20 years, and no available stage assessment during follow-up. Anyone involved in the research agrees to participate and agrees to have details the results of the research about them published. The flowchart for this process is presented in Figure 1. For the data analysis, 20,782 patients were finally enrolled.

### 2.2. The Definition and Diagnosis of Each Cirrhotic Complication

The diagnosis of esophageal varices (EVs), EV bleeding (EVB), ascites, sepsis, HE, or SBP was primarily based on individual ICD-9 or ICD-10 codes, shown in Supplementary Table S1. In addition, medications such as lactulose, or text mining of examinations such as upper esophagogastroduodenoscopy and abdominal echography by the Statistical Analysis System (SAS) regular expression technique, were used as adjuncts to support the diagnosis.

### 2.3. The Stage/Status Models

The stages considered as the original five and the novel seven-stage classification are reported in Table 1. Patients are supposed to have higher mortality risk with a higher stage. In addition, a patient’s stage might change with time but they do not have to go through all stages and can skip some stages.

### 2.4. The Primary Endpoint and Follow-Up

The primary endpoint was defined as overall mortality during the long-term follow-up period. To simplify our analysis, we ignored liver transplantation (LT) as a competing risk event. Censored data until the date of transplant were accounted for, or it was defined as death if the patient expired during liver transplant surgery.

Follow-up time was defined as the interval starting from the time when a patient was firstly diagnosed with liver cirrhosis until he or she died or underwent LT, or until the last medical record during the study period.

### 2.5. Covariates

Age, sex, etiologies of cirrhosis, and the Charlson comorbidity index (CCI) [24,25] were also used to assess the impact of the outcome of interest. The distribution of each item in the CCI is in Supplementary Table S2.

### 2.6. Statistical Methods

In descriptive statistics, continuous variables are expressed as mean ± standard deviation (SD) or median and interquartile range (IQR, 25–75 percentile) as appropriate, and categorical variables as frequencies and percentages (Table 2). The incidence rates of death in each of the five and seven stages as well as each range of the MELD are also presented in the results table.

Time-dependent receiver operating characteristic (ROC) curve analysis was used to assess the performance of the original five-stage model and the seven-stage model, as well as the MELD score without any variable adjustment. The predictive accuracy of death at time points of 3-months, 1-year, and 5-year overall survival was reported.

Multivariable Cox regression models with the stepwise model selection technique were built up to estimate the hazard ratio (HR) in the three different scoring systems. Harrell’s concordance index (C-index) for each model was calculated for assessing the prediction performance. The nomogram is representative of the formula of the model. Statistical analyses were performed by SAS version 9.4 (SAS Institute, Cary, NC, USA) and RStudio. A *p*-value of <0.05 was considered statistically significant.

Due to the issue of whether a patient’s cirrhotic stage could be reversed, especially in the decompensated stage, we performed sensitivity analysis for our proposed seven-stage prognostic system considering three different conditions: Condition ①: a patient’s stage could either halt, progress, or be reversed. Condition ②: a patient’s cirrhotic stage could only halt or progress. Condition ③: exclusion of patients with any progress then reversion of stages. The result of Condition ① was taken as the main result as it generally seemed closer to the reality.

## 3. Results

### 3.1. Flowchart and Demographics

In multi-institutional CGMH medical records, 43,638 patients with liver cirrhosis (LC) during 2007–2016 were included. Patients who were excluded included 13,399 who had a previous diagnosis of HCC before enrollment, 647 of age < 20 years, and 8810 without complete records for one or more relevant data points of interest. Finally, 20,782 patients were enrolled in this study (Figure 1). Table 2 shows demographic and clinical features of liver cirrhosis patients. The mean age was 56.58 and about 67.82% were male. Median follow-up time was 67.1 months. Of these study patients, 4427 (21%) died and 889 (4.28%) underwent liver transplant surgery.

### 3.2. The Incidence Rates of Death (Person-Years) for Each Clinical Stage and MELD Score

Table 3 shows the incidence rates of death for each stage. It increases with stage progression in the seven-stage clinical score, from 1.5% to 9.1%. Incidence of death exceeds 10% when the MELD score is over 25.

### 3.3. Prediction Power of Models

**Figure 2:**
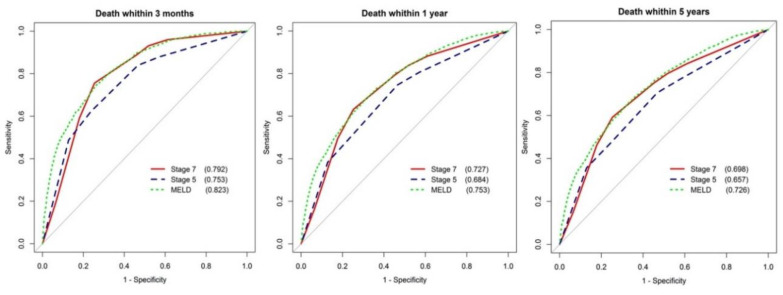
Comparison of the areas under the receiver operating characteristic (AUROC) of the five-stage and seven-stage models, as well as the MELD at 3 months, one year, and the entire follow-up period (5 years).

According to time-dependent ROC analysis (Figure 2), the seven-stage clinical score has a significantly better survival prediction than the original five-stage model by 4~5% (*p* < 0.001). However, the prediction performance of the seven-stage clinical score and MELD system are likely equivalent, with only about a 3% difference on average. Generally speaking, the area under ROC curves decreased with time, about a 10% decrease in prediction on average from 3 months to 5 years. As the predictive performance of the MELD-Na is equivalent to the MELD, for clarity and simplicity, the MELD was chosen in further model analysis.

### 3.4. Cox Model Analysis

The collected clinical variables considered in univariate Cox regression analysis are reported in Supplementary Table S3 for reference. Three predictive models adjusted by age and CCI are shown in Table 4. The result shows that the seven clinical stages are associated with an increased mortality risk in general. Although the estimated hazard ratio of stage 2 to stage 1 was not significant (HR: 0.98, *p* = 0.839), afterward, the hazard ratio became increasingly different from 1.21 (stage 3) to 4.25 (stage 7). However, no monotone increasing of hazard ratio was found in the five-stage model. According to the C-index, Model III with the MELD score has the highest prediction performance (C-index = 0.797) of all (Table 4).

### 3.5. The Nomograms of the Prognostic Indexes

**Figure 3:**
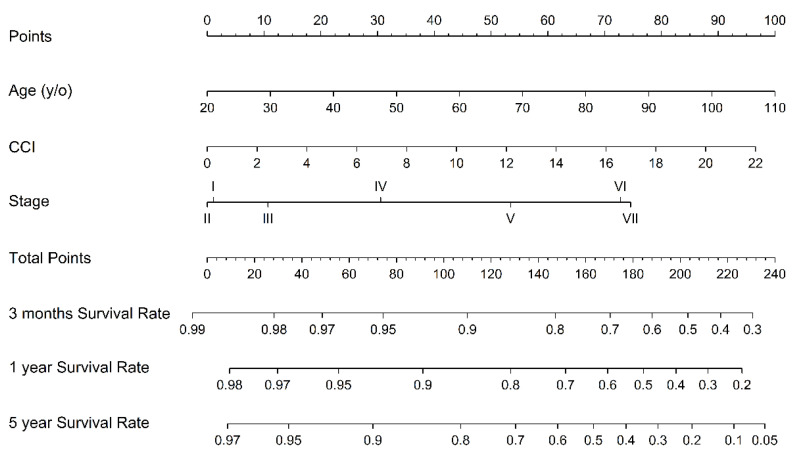
The nomograms of the prognostic indexes based on age, CCI (Charlson comorbidity index), and seven-stage clinical score. They give an estimate of the expected survival.

**Figure 4:**
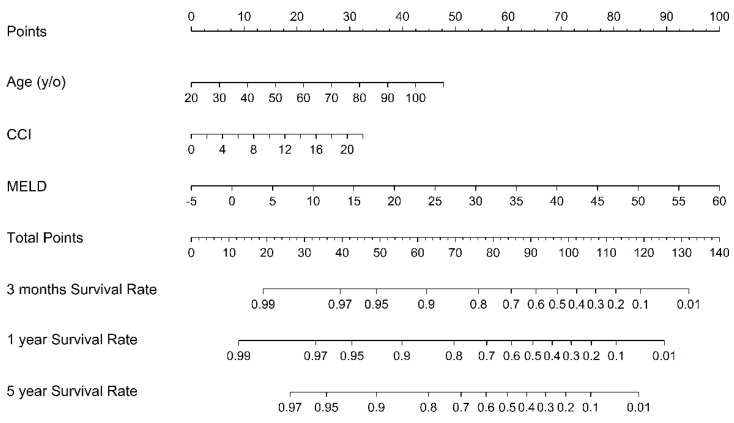
The nomograms of the prognostic indexes based on age, CCI, and the MELD (Model for End-Stage Liver Disease). They give an estimate of the expected survival.

Figure 3 shows the nomograms of our proposed seven-stage clinical score, reflecting the instant death prediction for reference. Furthermore, the MELD predictive value increases with age and CCI. Nomograms are representative of the formula of the model (Figure 4).

A proposed on-line prognostic index based on age, CCI, and clinical stage or the MELD was also implemented for physicians’ and patients’ reference (On-line prognostic index based on age, CCI, and clinical stage or the MELD. Available online: https://clinical-meld-scores-cgrd.000webhostapp.com/ (accessed on 19 October 2020)).

### 3.6. Sensitivity Analysis

Sensitivity analysis for the issue of whether the cirrhotic stage is reversible showed similar results of increasing hazard ratio of mortality with more advanced clinical stage during long-term follow-up, which was noted in both Condition ② and Condition ③. The estimates were very close to those in the model of Condition ①, assuring robust results. The results are presented in Supplementary Table S4.

## 4. Discussion

Our study proved that the implementation of a novel accurate clinical assessment of cirrhosis, considering seven clinical events, has produced a good prognostic tool. Definitely in our analysis, it was better than a classification based on five stages, which is currently still accepted [26]. In addition, the study proved that the MELD criteria maintain their high validity, as well as versus accurate clinical assessment.

The assessment of cirrhosis stages on the basis of simple clinical findings seems to be efficient, just like the New York Heart Association functional classes for patients with heart failure [27]. It provides easily obtainable information from medical histories and physical examination that stratify patients according to their short-term risk of death.

The advantages of the seven-stage model include raising patients’ awareness of their illness, patient–physician interactions, and educational programs. It is an alternative tool to the ones based on biochemical determinations, such as the MELD, because biochemical data require at least one hour to be available [28]. An on-line prognostic index based on age, CCI, clinical stage or the MELD was also implemented (On-line prognostic index based on age, CCI, and clinical stage or the MELD. Available online: https://clinical-meld-scores-cgrd.000webhostapp.com/ (accessed on 19 October 2020)) for physicians’ and patients’ reference.

The study also confirmed the high prognostic validity of the MELD not only at three months, but also on a longer perspective in which it has been less validated and the optimal allocation strategy based on scoring systems is still under debate [13]. Furthermore, it showed that even an accurate assessment based on clinical events cannot substitute the MELD scoring system to assess the prognosis of patients, even if it may be useful to integrate it, as shown in Table 4.

The reasons why this seven-stage prognostic model was better than the five-stage model depend on the information nested in the occurrence of HE or SBP, which were not categorized in the five-stage model (Supplementary Table S5). In addition, two studies supported our seven-stage model in terms of changing EVB in stage 3 and ascites in stage 4. One study used a four-stage model in patients with low MELD scores (≤20) awaiting liver transplantation to help select candidates for more aggressive monitoring or extended criteria donation [29]. The researchers placed ascites rather than bleeding varices in stage 4 and found this model helpful. In another study, ascites was found to be a better stratifying clinical event than variceal hemorrhage in patients with decompensated cirrhosis [30]. In our seven-stage model, EVB was set to stage 3 due to a hazard ratio for primary endpoints in a competing risk regression analysis of 1.93. Meanwhile, ascites was 2.19, hence it was placed in stage 4. These results may reflect the improvement in the management of variceal hemorrhage [21].

Furthermore, many studies supported the use of HE and SBP in the prognostic model. West Haven grade 3–4 HE at the time of waiting list registration eminently increased 90-day waiting list mortality independent of MELD scores [31]. Severe hepatic encephalopathy accounted for a mortality of more than 50% in the first year alone [32]. One study showed that the addition of an automatically obtained electroencephalographic (EEG)-based index improves the prognostic accuracy of the MELD score [33]. Therefore, incorporating HE in the assessment of LT priority might improve prognosis of liver disease severity and prioritization for LT [31]. Further, SBP is a major and severe complication in cirrhosis patients with ascites. One-year overall mortality rates were as high as 78% [34]. Thus, liver transplantation should be seriously considered for survivors of SBP who are otherwise good transplantation candidates [35].

Our study has some limitations. First, the information obtained by the speed of occurrence of decompensation could not be analyzed, thus the role of acute or chronic liver failure could not be distinguished. Second, the role of etiological treatment, such as alcohol abstinence and antiviral drugs, could not be properly incorporated into the study, since it had begun before the present treatments were developed. Third, we could not differentiate the patients with diuretic-sensitive ascites from those with refractory ascites, because of the features of our database, which refers to the ICD-9/10 code. However, randomized trials have shown that less than 10% of patients with cirrhosis and ascites are refractory to standard medical therapy [36] and patients with refractory ascites are prone to the development of SBP [37], and hence would be classified into the final 7th stage. This might minimize the survival difference between these two subgroups in patients who remain classified in the ascites stage. Fourth, using ICD-9-CM codes for patient screening carries the potential risk of overlooking participants due to coding errors. However, when ICD-9-CM codes were matched with hospital electronic medical records in other validation studies, the international classification of diseases (ICD) codes showed a sensitivity of up to 99% for positive predictive value against gold standard electronic medical records [38]. Fifth, the proportion of hepatitis B virus (HBV)-related cirrhosis may have been underestimated given that some patients had delayed hepatitis B surface antigen seroclearance before 2006 and hence HBsAg was negative during serial follow-up. As such, these cases could be attributed to other etiologies if diagnostic coding was incorrect. At any rate, etiology did not have a relevant role in the model. Sixth, the definition of sepsis in this study was primarily based on the ICD codes. A prospective study focused on this item might produce more accurate results. Seventh, at the patient enrollment stage, we did not retrieve information about whether patients with non-B non-C non-alcoholic cirrhosis had hepatitis A virus (HAV) or hepatitis E virus (HEV) infection because we thought HAV or HEV are an acute infections that are usually self-limiting, and rarely cause chronic liver disease unless in an immunocompromised status (e.g., patients receiving solid-organ transplants or who are human immunodeficiency virus (HIV) positive). Eighth, the baseline MELD scores of 43.61% of the patients were not available because their serum Creatinine (Cr), bilirubin, and INR values were not measured at the same time and hence were not analyzed. The detailed missing data are reported in Supplementary Table S5. However, the large patient cohort could compensate for this. Eighth, validation of the seven-stage model by the Markov model is warranted in further study by extending our data. Despite these limits which suggest the opportunity of a prospective multicenter validation, this study has the merit to have enrolled a huge number of subjects and, thus, provides solid information to be shared.

## 5. Conclusions

Our study has proved that accurate clinical staging of cirrhosis based on seven stages has higher prognostic value than the five-stage model, and confirmed the validity of the MELD vs. clinical assessment. Based on the above cited models, we have provided an estimate of survival for a large group of liver cirrhosis patients.

## Figures and Tables

**Table 1:** The original five-stage and proposed seven-stage models.

Original Five-Stage Prognostic System ^#^	Interpretation
Compensated LC:	
Stage 1 (no complication)	EV−, EVB−, Ascites−, Sepsis−
Stage 2 (EV)	EV+; EVB−, Ascites−, Sepsis−
Decompensated LC	
Stage 3 (ascites)	Ascites+, EV±, EVB−, Sepsis−
Stage 4 (EVB)	EV+ & EVB+; Ascites±; Sepsis−
Stage 5 (sepsis)	Sepsis+, EV±, EVB±, Ascites±
**Innovated Seven-Stage Prognostic System**	**Interpretation**
Compensated LC	
Stage 1 (no complication)	Without complication and CTP score ≤ 6
Stage 2 (EV)	EV+; EVB−; Ascites−; Sepsis−; HE−; SBP−
Decompensated LC	
Stage 3 (EVB)	EVB+; EV±; Ascites−; Sepsis−; HE−; SBP−
Stage 4 (ascites)	Ascites+; EV±; EVB±; Sepsis−; HE−; SBP−
Stage 5 (sepsis)	Sepsis+; EV±; EVB±; Ascites±; HE−; SBP−
Stage 6 (HE)	HE+; EV±; EVB±; Ascites±; Sepsis±; SBP−
Stage 7 (SBP)	SBP+; EV±; EVB±; Ascites±; Sepsis±; HE±

^#^ The original five-stage prognostic system (varices, ascites, variceal bleeding, and sepsis) was proposed by D’Amico (18) and Arvaniti et al. (19); +: Presence; −: Absence; LC: Liver cirrhosis; EV: Esophageal varices; EVB: Esophageal variceal bleeding; HE: Hepatic encephalopathy; SBP: Spontaneous bacterial peritonitis.

**Table 2:** Demographics of 20,782 cirrhotic patients.

Variable	Statistics
Age	56.58 ± 14.72
Sex	
Male	14,095 (67.82)
Female	6687 (32.18)
Etiologies of LC *	
Hepatitis B	6928 (33.33)
Hepatitis C	3114 (14.98)
Alcoholic liver	2409 (11.59)
Non-B/C/ALC	8326 (40.09)
Biochemistry	
Creatinine (Cr), mg/dL	0.82 (0.64–1.11)
Na, mEq/L	139 (136–141)
alanine aminotransferase (ALT), U/L	36 (22–66)
aspartate aminotransferase (AST), U/L	52 (32–92)
Bilirubin Total, mg/dL	1.2 (0.7–2.4)
Albumin, g/dL	3.2 (2.6–3.87)
Hemogram	
White blood cells (WBC), ×1000/µL	5.9 (4.2–8.2)
International normalized ratio (INR)	1.2 (1.04–1.4)
Platelet (PLT), ×1000/µL	118 (71–197)
Clinical Index	
Model for End-Stage Liver Disease, MELD score	11.38 (7.55, 16.91)
Charlson comorbidity index (CCI)	4 (2–6)
Median follow-up time (months)	67.10 (32.59–102.18)
Outcome	
Mortality	4427 (21.30)
LT	889 (4.28)

Statistics are in terms of three types: Mean ± SD/Median (IQR)/percentage (%); * the actual LC etiology percentage may be biased given that this classification primary relies on serum tests such as HBsAg, anti-HCV antibody, ICD-9, ICD-10 diagnostic codes, and anti-hepatitis viral agents found in our Chang-Gung Research Database (CGRD). It does not include either hepatitis B virus (HBV)-DNA or hepatitis C virus (HCV)-RNA data and cannot be reviewed by clinicians. Thus, these results may underestimate the true percentage of HBV- or HCV-related cirrhosis in Taiwan. Non-B/C/ALC: Cirrhotic etiology not attributed to definite HBV, HCV, or alcohol. Most of them were probably non-alcoholic steatohepatitis (NASH) related. LT: Liver transplantation.

**Table 3:** The incidence rates of death (person-years) for each clinical stage and MELD score.

	Baseline	Follow-Up		
	N (%)	Number of Deaths	Total Years Observed	Incidence of Death (Person-Years)
Five-stage clinical score				
Compensated LC				
Stage 1 (no complication)	10,179 (48.98)	986	52,304.81	1.9%
Stage 2 (EV)	1609 (7.74)	187	7106.94	2.6%
Decompensated LC				
Stage 3 (ascites)	4235 (20.38)	907	19,890.90	4.6%
Stage 4 (EVB)	2199 (10.58)	426	10,106.92	4.2%
Stage 5 (sepsis)	2560 (12.32)	1216	14,695.32	8.3%
Seven-stage clinical score				
Compensated LC				
Stage 1 (no complication)	9265 (44.58)	730	47,899.30	1.5%
Stage 2 (EV)	1462 (7.03)	161	6250.69	2.6%
Decompensated LC				
Stage 3 (EVB)	1349 (6.49)	180	5851.21	3.1%
Stage 4 (ascites)	3831 (18.43)	637	17,365.39	3.7%
Stage 5 (sepsis)	1593 (7.67)	529	9609.12	5.5%
Stage 6 (HE)	2212 (10.64)	941	11,135.16	8.5%
Stage 7 (SBP)	1070 (5.15)	544	5994.03	9.1%
MELD score ^§^				
≤10	5126 (24.67)	426	26,244.01	1.6%
11~15	2825 (13.59)	522	13,206.03	4.0%
16~20	1525 (7.34)	405	7089.04	5.7%
21~25	1086 (5.23)	345	5009.86	6.9%
26~30	568 (2.73)	265	2609.55	10.2%
31~35	278 (1.34)	160	1194.09	13.4%
35~40	310 (1.49)	217	1192.69	18.2%

^§^ There was a lack of baseline MELD scores (only 11,718 (56.4%) valid cases).

**Table 4:** Multivariable Cox regression models for overall mortality.

Variable	Model I		Model II		Model III	
	aHR (95% C.I.)	*p*-Value	aHR (95% C.I.)	*p*-Value	aHR (95% C.I.)	*p*-Value
Age	1.02 (1.02–1.03)	<0.001	1.02 (1.02–1.02)	<0.001	1.03 (1.02–1.03)	<0.001
CCI	1.09 (1.08–1.10)	<0.001	1.10 (1.10–1.12)	<0.001	1.08 (1.07–1.10)	<0.001
MELD					1.06 (1.05–1.06)	<0.001
Stage #						
1						
2	0.98 (0.84–1.14)	0.8387	0.89 (0.78–1.01)	0.073		
3	1.21 (1.04–1.42)	0.0159	1.85 (1.70–2.01)	<0.001		
4	1.81 (1.64–2.00)	<0.001	1.40(1.25–1.56)	<0.001		
5	2.81 (2.52–3.15)	<0.001	3.45 (3.18–3.75)	<0.001		
6	4.11 (3.75–4.51)	<0.001				
7	4.25 (3.80–4.74)	<0.001				
	C-Index = 0.751		C-Index = 0.727		C-Index = 0.797	

# According to multivariable models, the estimated hazard ratio increased with the stage except for stage 2 compared with stage 1. The others were statistically significant. Adjusted hazard ratio (aHR) for multiple Cox model for overall survival.

## Supplementary Materials

The following are available online at https://www.mdpi.com/2075-4426/10/4/186/s1, Table S1: The definition and diagnosis code of each cirrhotic complication. Table S2: The distribution of each Charlson Comorbidity Index (CCI) items. Table S3: Univariate Cox regression analysis Table S4: Multivariable Cox and competing-risk regression analysis for the 5-year survival in Condition ② model Multivariable Cox and competing-risk regression analysis for the 5-year survival in Condition ③ model. Table S5: Details of the valid and missing data.
