# Supplementary material for: Hepatic Encephalopathy and Spontaneous Bacterial Peritonitis Improve Cirrhosis Outcome Prediction: A Modified Seven-Stage Model as a Clinical Alternative to MELD
Source: J Pers Med. 2020 Oct 22;10(4):186. doi: 10.3390/jpm10040186 (PMC7711993; doi:10.3390/jpm10040186)
Supplement: Supplementary file 1 [file jpm-10-00186-s001.pdf]

Supplementary Table 1. The definition and diagnosis code of each cirrhotic complication.

Cirrhotic complication	ICD-9-CODE	ICD-10-CODE
**EV without bleeding**	45621,4561	I8500, I8510
**EV with bleeding**	45620, 4560, 53082	I8511, I8501
**Ascites**	7895	R18, K7031, K7151
**Sepsis**	7907, 038, 99591,99592, 0031	R7881, A021, A40, A41, B377, R652, A32, A5486
**HE**	3483, 5722	G934
**SBP**	56723, 56729, 5679, 5678,5670	K652

EV: Esophageal varices. HE: Hepatic encephalopathy. SBP: Spontaneous bacterial peritonitis.

Supplementary Table 2. The distribution of each Charlson Comorbidity Index (CCI) items.

CCI items	Count	%
Myocardial Infarction	330	1.59
Congestive Heart Failure	1,505	7.24
Periphral Vascular Disease	368	1.77
Cerebrovascular Disease	1,996	9.60
Dementia	462	2.22
Chronic Pulmonary Disease	2,176	10.47
Rheumatic Disease	194	0.93
Ulcer Disease	7,721	37.15
Diabetes without complications	5,841	28.11
Diabetes with complications	1,430	6.88
Paraplegia and Hemiplegia	140	0.67
Renal Disease	1,918	9.23
Cancer (Any malignancy, including leukemia and lymphoma)	6,323	30.43
Moderate or severe liver disease	11,517	55.42
Metastatic Carcinoma	947	4.56
AIDS/HIV	12	0.06

Supplementary Table 3. Univariate Cox regression analysis.

Variable	HR (95% CI)		p-value
**Age**	1.029	(1.027 – 1.031)	<0.001
Sex (F vs. M)	1.071	(1.005 – 1.142)	0.035
Hepatitis B (Y vs. N)	0.969	(0.912 – 1.030)	0.312
Hepatitis C (Y vs. N)	1.104	(1.032 – 1.180)	0.004
Alcoholic liver (Y vs. N)	1.351	(1.122 – 1.607)	0.001
Cr, mg/dL	1.186	(1.173 – 1.199)	<0.001
Na	0.993	(0.991 – 0.995)	<0.001
ALT	1.000	(1.000 – 1.000)	<0.001
AST	1.000	(1.000 – 1.001)	<0.001
Bilirubin Total, mg/dL	1.055	(1.052 – 1.059)	<0.001
Albumin, g/dL	0.575	(0.555 – 0.596)	<0.001
WBC	1.048	(1.045 – 1.051)	<0.001
INR	1.546	(1.509 – 1.582)	<0.001
PLT	0.997	(0.996 – 0.997)	<0.001
MELD score	1.080	(1.076 – 1.083)	<0.001
ALBI score	1.836	(1.773 – 1.902)	<0.001
CCI (Charlson Comorbidity Index)	1.154	(1.144 – 1.165)	<0.001
Stage 7*			
1	1 (reference)		
2	1.197	(1.028 – 1.389)	0.019
3	1.411	(1.204 – 1.646)	<0.001
4	2.190	(1.981 – 2.419)	<0.001
5	3.240	(2.891 – 3.628)	<0.001
6	5.169	(4.725 – 5.660)	<0.001
7	4.886	(4.378 – 5.451)	<0.001
Stage 5*			
1	1 (reference)		
2	1.077	(0.943 – 1.226)	0.268
3	2.229	(2.053 – 2.421)	<0.001
4	1.638	(1.469 – 1.824)	<0.001
5	3.970	(3.658 – 4.309)	<0.001

Supplementary Table 4. Multivariable Cox and competing-risk regression analysis for the 5-year survival in Condition ② model[$]. Multivariable Cox and competing-risk regression analysis for the 5-year survival in Condition③ model[&].

**Cox model**	**Condition ①**		**Condition ②**		**Condition ③**	
	**aHR (95% C.I.)**	**p-value**	**aHR (95% C.I.)**	**p-value**	**aHR (95% C.I.)**	**p-value**
**Age**	1.02 (1.02 – 1.03)	<0.001	1.02 (1.02-1.02)	<0.001	1.02 (1.016-1.022)	<0.001
**CCI**	1.09 (1.08 – 1.10)	<0.001	1.09 (1.08-1.10)	<0.001	1.09 (1.079-1.102)	<0.001
**7-Stage**						
1	1 (reference group)		1 (reference group)		1 (reference group)	
2	0.98 (0.84 – 1.14)	0.8387	1.09 (0.91-1.31)	0.3288	1.13 (0.93-1.37)	0.2115
3	1.21 (1.04 – 1.42)	0.0159	1.45 (1.22-1.72)	<0.001	1.52 (1.25-1.84)	<0.001
4	1.81 (1.64 – 2.00)	<0.001	1.94 (1.74-2.17)	<0.001	1.94 (1.72-2.18)	<0.001
5	2.81 (2.52 – 3.15)	<0.001	3.30 (2.93-3.72)	<0.001	3.64 (3.20-4.13)	<0.001
6	4.11 (3.75 – 4.51)	<0.001	4.93 (4.47-5.44)	<0.001	5.02 (4.53-5.58)	<0.001
7	4.25 (3.80– 4.74)	<0.001	5.91 (5.32-6.577)	<0.001	5.28 (4.66-5.98)	<0.001

CRR	Condition ①		Condition ②		Condition ③	
	**sHR (95% C.I.)**	**p-value**	**sHR (95% C.I.)**	p-value	**sHR (95% C.I.)**	p-value
**Age**	1.02 (1.02 – 1.02)	<0.001	1.02 (1.01-1.02)	<0.001	1.02 (1.01-1.02)	<0.001
**CCI**	1.08 (1.07 – 1.09)	<0.001	1.08 (1.07-1.09)	<0.001	1.08 (1.07-1.09)	<0.001
**7-Stage**						
1	1 (reference group)		1 (reference group)		1 (reference group)	
2	1.46 (1.26 – 1.70)	<0.001	1.31 (1.09-1.56)	0.003	1.31 (1.09-1.59)	0.005
3	1.93 (1.66 – 2.26)	<0.001	1.85 (1.57-2.18)	<0.001	1.87 (1.55-2.26)	<0.001
4	2.19 (1.99 – 2.42)	<0.001	2.08 (1.87-2.31)	<0.001	2.05 (1.83-2.29)	<0.001
5	2.70 (2.42 – 3.02)	<0.001	2.76 (2.46-3.09)	<0.001	2.85 (2.52-3.22)	<0.001
6	3.37 (3.08 – 3.69)	<0.001	3.57 (3.24-3.93)	<0.001	3.50 (3.16-3.88)	<0.001
7	4.22 (3.78 – 4.70)	<0.001	4.51 (4.07-4.99)	<0.001	4.25 (3.76-4.81)	<0.001

sHR: subhazard ratio. [$] Condition 2 model presented in which a patient's cirrhotic stage could only halt on or progress. [&] Condition 3 model presented in which patients with any progress then reverse of stages were excluded. Sub-Hazard Ratio (SHR) for competing risk regression analysis: primary endpoint: mortality, competing-risk event: liver transplantation.

Supplementary Table 5. Details of the valid and missing data.

Variable	Statistics	valid N	missing %
**Age**	56.58 ±14.72	20782	0.00%
**Sex**		20782	0.00%
Male	14,095(67.82)		
Female	6,687(32.18)		
**Etiologies of LC***		20782	0.00%
Hepatitis B	6,928 (33.33)		
Hepatitis C	3,114 (14.98)		
Alcoholic liver	2,409 (11.59)		
Non-B/C/ALC	8,326(40.09)		
**Biochemistry**			
Cr, mg/dL	0.82 (0.64 – 1.11)	17429	16.13%
Na	139 (136 – 141)	16261	21.75%
ALT	36 (22 – 66)	17097	17.73%
AST	52 (32 – 92)	17016	18.12%
Bilirubin Total, mg/dL	1.2 (0.7 – 2.4)	17821	14.25%
Albumin, g/dL	3.2 (2.6 – 3.87)	16068	22.68%
**Hemogram**			
WBC	5.9 (4.2 – 8.2)	17102	17.71%
INR	1.2 (1.04– 1.4)	16140	22.34%
PLT	118 (71 – 197)	17103	17.70%
**Clinical Index**			
MELD score	11.38 (7.55, 16.91)	11718	43.61%
CCI (Charlson Comorbidity Index)	4 (2 – 6)	20782	0.00%
**Median Follow time (months)**	67.10(32.59-102.18)	20782	0.00%
**Outcome**		20782	0.00%
Mortality	4,427(21.30)		
LT	889 (4.28)		
